# Wastewater Surveillance Pilot at US Military Installations: Cost Model Analysis

**DOI:** 10.2196/54750

**Published:** 2024-09-06

**Authors:** Jaleal S Sanjak, Erin M McAuley, Justin Raybern, Richard Pinkham, Jacob Tarnowski, Nicole Miko, Bridgette Rasmussen, Christian J Manalo, Michael Goodson, Blake Stamps, Bryan Necciai, Shanmuga Sozhamannan, Ezekiel J Maier

**Affiliations:** 1 Booz Allen Hamilton, 4747 Bethesda Ave, Bethesda, MD, United States, 1 5712413499; 2 United State Air Force Research Laboratory, Wright Patterson Air Force Base, OH, United States; 3 Chemical, Biological, Radiological and Nuclear Defense Enabling Biotechnologies, Joint Program Executive Office for Chemical, Biological, Radiological and Nuclear Defense, Frederick, MD, United States; 4 Joint Research and Development, Inc, Stafford, VA, United States

**Keywords:** wastewater surveillance, cost analysis, military health, public health, sanitation, sanitary, water, wastewater, surveillance, environment, environmental, cost, costs, economic, economics, finance, financial, pathogen, pathogens, biosurveillance

## Abstract

**Background:**

The COVID-19 pandemic highlighted the need for pathogen surveillance systems to augment both early warning and outbreak monitoring/control efforts. Community wastewater samples provide a rapid and accurate source of environmental surveillance data to complement direct patient sampling. Due to its global presence and critical missions, the US military is a leader in global pandemic preparedness efforts. Clinical testing for COVID-19 on US Air Force (USAF) bases (AFBs) was effective but costly with respect to direct monetary costs and indirect costs due to lost time. To remain operating at peak capacity, such bases sought a more passive surveillance option and piloted wastewater surveillance (WWS) at 17 AFBs to demonstrate feasibility, safety, utility, and cost-effectiveness from May 2021 to January 2022.

**Objective:**

We model the costs of a wastewater program for pathogens of public health concern within the specific context of US military installations using assumptions based on the results of the USAF and Joint Program Executive Office for Chemical, Biological, Radiological and Nuclear Defense pilot program. The objective was to determine the cost of deploying WWS to all AFBs relative to clinical swab testing surveillance regimes.

**Methods:**

A WWS cost projection model was built based on subject matter expert input and actual costs incurred during the WWS pilot program at USAF AFBs. Several SARS-CoV-2 circulation scenarios were considered, and the costs of both WWS and clinical swab testing were projected. Analysis was conducted to determine the break-even point and how a reduction in swab testing could unlock funds to enable WWS to occur in parallel.

**Results:**

Our model confirmed that WWS is complementary and highly cost-effective when compared to existing alternative forms of biosurveillance. We found that the cost of WWS was between US $10.5-$18.5 million less expensive annually in direct costs as compared to clinical swab testing surveillance. When the indirect cost of lost work was incorporated, including lost work associated with required clinical swab testing, we estimated that over two-thirds of clinical swab testing could be maintained with no additional costs upon implementation of WWS.

**Conclusions:**

Our results support the adoption of WWS across US military installations as part of a more comprehensive and early warning system that will enable adaptive monitoring during disease outbreaks in a more cost-effective manner than swab testing alone.

## Introduction

Many human pathogens are shed within bodily fluids during active infection and make their way into the domestic sanitary sewage system along several routes. Therefore, wastewater sample collection is a viable approach to monitor the prevalence of pathogens [1], including those of pandemic potential and biodefense/biosecurity relevance. Early in the COVID-19 pandemic, researchers identified that SARS-CoV-2 RNA was shed into fecal matter at viral loads high enough to be detected in wastewater [2-4]. Therefore, the preexisting field of wastewater-based epidemiology rallied to transition preexisting methods [5] from academic research into scalable public health surveillance tools. Especially early in the COVID-19 pandemic, traditional swab-based testing could not scale quickly enough to serve as a reliable source of population-level disease transmission data [5-7]. Multiple studies explored the efficacy of wastewater surveillance (WWS) as a stream of epidemiological data to complement case tracking for community transmission monitoring, finding that WWS data tracks trends in clinical case reporting data [8-10]. While the statistical correlation between viral load in sewage and clinical indicators is strong, the exact quantitative relationship between individual-level testing and WWS data is complex and depends on a variety of factors, including the epidemiology of the outbreak, data collection, and processing timelines [11]. Despite these complexities, WWS has been shown to be correlated with community infection dynamics [10] in addition to simply being an effective qualitative detection tool. Implementing WWS within institutional building complexes, such as college campuses, has unique challenges but also enables building-level resolution monitoring and early warning capabilities [12-15]. WWS can be a leading qualitative indicator of disease presence in a community when overall disease prevalence is low, making WWS a good candidate for broad-scale baseline pathogen monitoring [16]. Because WWS is passive and independent of health care seeking behavior, it provides a data stream complementary to active tracking of infections or hospitalizations, both of which have limitations. Additional benefits of WWS include the ability to monitor multiple pathogens, emerging viral variants, and nonbiological hazards [17].

The US Government prioritized WWS to track the spread of COVID-19 and other diseases. For example, environmental monitoring for viral threats via WWS is a key component of pandemic threat early warning systems prioritized in the Biden administration’s “American Pandemic Preparedness: Transforming our Capabilities” plan [18]. In addition, the Under Secretary of Defense for Personnel and Readiness directed the US Department of Defense (DoD) to leverage alternative technologies, including WWS, to supplement existing surveillance strategies in a memorandum titled “Consolidated Department of Defense Coronavirus Disease 2019 Force Health Protection Guidance” [19]. The Centers for Disease Control and Prevention established the National Wastewater Surveillance System [20] to broaden traditional diagnostic test surveillance systems by enabling efficient collection of community-level samples. In addition, the Centers for Disease Control and Prevention is applying WWS within passenger airplanes as part of its Traveler Genomic Surveillance program [21]. Finally, in June 2021, the National Institute of Standards and Technology and the Department of Homeland Security, Science and Technology Directorate convened a virtual workshop entitled “Standards to Support an Enduring Capability in Wastewater Surveillance for Public Health” to identify challenges and solutions for maturing and ensuring WWS capabilities for detecting and monitoring public health threats [22,23].

As a result of practical successes in early research and implementation studies, best practices emerged for how to implement WWS at scale [6]. WWS can be an important tool for epidemiological monitoring and outbreak response if implemented with consideration for various challenges [24,25]; one important aspect to consider is avoiding redundancy with clinical testing by implementing a joint surveillance strategy. The design of a WWS data collection scheme and methods of analysis can have significant impacts on bias and interpretation of the data [26]. When implemented according to best practices, WWS can be a cost-effective part of a public health response system [27]. Pairing WWS with clinical testing allows for both approaches to serve specific needs, thereby enhancing the cost-effectiveness of both [28].

The DoD has installations around the globe with small compact living communities, some of which have overlapping sewersheds with nearby cities. Tens of thousands of military personnel and civilians live and work in these installations. The DoD implements a four-tiered COVID-19 testing scheme. The first three tiers focus on staff at varying levels of critical service and deployment; tier 4 sentinel surveillance is an asymptomatic testing program designed to cover all personnel. Therefore, we focused on tier 4 sentinel surveillance as our point of comparison for WWS cost since WWS is also best suited for broad population monitoring.

Similarities exist between DoD installations and other institutional building complexes like college campuses. Yet implementing a WWS system at DoD sites requires special planning considerations given unique operational constraints and global scale. To address these issues, the DoD commissioned several WWS pilot studies aimed at figuring out the logistical, operational, and financial aspects of implementing a WWS program. One such study demonstrated the effectiveness of wastewater screening of blackwater from Coast Guard vessels [29]. Another study focused on WWS at air force bases (AFBs); the US Air Force (USAF) and Joint Program Executive Office for Chemical, Biological, Radiological and Nuclear Defense (JPEO-CBRND) WWS pilot study was larger than previous DoD pilots and more representative of US military installations globally. We analyze the cost-effectiveness of WWS within the DoD context, based on the results from the USAF and JPEO-CBRND WWS pilot study. We developed a cost model that includes upfront capital expenditures, operational expenditures, and indirect costs of lost work time. Further, we performed a break-even analysis to explore how traditional swab testing and WWS could be carried out in tandem within the budget of existing swab testing schemes. We concluded that WWS is cost-effective as a complementary passive community-level disease surveillance scheme, within the context of AFBs, and therefore likely would be cost-effective as a DoD-wide global multi-pathogen monitoring system that could be operated in complement to swab-based testing in the event of future disease outbreaks.

## Methods

### WWS Pilot Study Design

To assess the feasibility of WWS for SARS-CoV-2 within the USAF context, a multidisciplinary working group was assembled, and a pilot scale implementation was organized. The effort was also coordinated with DoD partners through collaboration with the Office of the Assistant Secretary of Defense for Health Affairs and JPEO-CBRND. A total of 26 AFBs were first contacted for enrollment via an invitation from a public health emergency officer or a USAF air staff logistics directorate of civil engineers memo, and all 26 sites expressed interest. WWS was ultimately piloted at 17 AFBs to demonstrate feasibility, safety, utility, and cost-effectiveness from May 2021 to January 2022. During the initial phase, conducted from June through August 2021, WWS techniques were deployed for testing at three remote sites. Next, WWS was evaluated at a larger scale, with 14 additional sites executing standardized procedures to collect and process wastewater samples once per week. The project utilized a portable quantitative polymerase chain reaction (PCR) instrument (Biomeme) [27] and a digital PCR (dPCR). AFB site personnel were trained to identify detectable SARS-CoV-2 using both systems. In addition, a passive sampling device was prototyped to decrease costs associated with expensive autosampler procurement.

### Collection of Tier 4 Sentinel Surveillance and WWS Costs

We gathered known costs or made estimates of direct costs for all activities required to implement both tier 4 diagnostic testing and WWS protocols. The costs of WWS were based on actual material costs and levels of effort from the WWS pilot study. Costs of tier 4 diagnostic testing were based directly on USAF experience. Costs included fixed and variable equipment and material costs, the costs of USAF labor (salaries and benefits), and estimated fully loaded contractor labor (billing) rates. Specific cost parameter values and sources are shown in Table 1.

**Table 1.** Cost analysis model parameters, with the values used and the sources of the values.

Parameter		Value	Source
**Tier 4 parameters**			
	PCR^a^ tests (nonoutbreak)	293	Tier 4 PCR surveillance plan (low end of estimate)
	PCR tests (outbreak)	586	Tier 4 PCR surveillance plan (high end of estimate)
	Testing time (minutes per patient)	30	Estimate
	Sample time (laboratory technician minutes)	10	Estimate
	Sample time (nurse minutes)	5	Estimate
	Data management and reporting (nurse minutes)	5	Estimate
	Data management and reporting (laboratory technician minutes)	2	Estimate
	Benefits adjustment to salaries (%)	35	Common practice; consistent with Bureau of Labor Statistics data
	Nurse average salary including benefits (US $)	87,750	[28]
	Laboratory technician average salary including benefits (US $)	55,687.50	[29]
	Average USAF^b^ salary including benefits (US $)	81,000	[30]
	Material cost per PCR test (US $)	50	DoD^c^ SME^d^
	Total cost per PCR test (including labor; US $)	62.39	Estimate: material cost of a PCR test plus the cost of labor per PCR test
**WWS^e^ parameters**			
	Wastewater tests per month (nonoutbreak)	4.33 (1/week)	Phase 2 testing cadence
	Wastewater tests per month (outbreak)	21.66 (5/week)	Estimate
	Android device cost (US $)	600	DoD SME/vendor
	Thermocycler cost (US $)	9950	DoD SME/vendor
	Cooker and cooking container cost (US $)	271.88	DoD SME/vendor
	Biomeme sample preparation tray cost (US $)	200	DoD SME/vendor
	DynaMag-50 magnet cost (US $)	960	DoD SME/vendor
	M1 sample prep cartridge kit (cost per test; US $)	45	DoD SME/vendor
	Go strips (cost per test; US $)	300	DoD SME/vendor
	Materials cost per wastewater test (US $)	54.41	Vendor
	Supply shipping costs (boxes; US $)	22.66	DoD SME
	Supply shipping costs (FedEx; US $)	34.66	DoD SME
	Labor hours per test	6	Air Force and Booz Allen estimate
	Hourly wage of sampler (US $)	13.10	Airforce.com
	Base selection costs (per base; US $)	2050.40	Technical and pricing SME for pilot study activities
	Base onboarding and training costs (per base; US $)	2184.93	Technical and pricing SME for pilot study activities
	Surveillance, data management, and reporting costs (per base per week; US $)	265.35	Technical and pricing SME for pilot study activities
	Ongoing support costs (per base per week; US $)	87.95	Technical and pricing SME for pilot study activities
	Vendor management costs (per base per week; US $)	90.29	Technical and pricing SME for pilot study activities
	Program coordination and oversight costs (per base per week; US $)	105.53	Technical and pricing SME for pilot study activities

a PCR: polymerase chain reaction.

b USAF: US Air Force.

c DoD: Department of Defense.

d SME: subject matter expert.

e WWS: wastewater surveillance.

Materials costs modeled for tier 4 swab testing only include the total cost of the PCR swab test, which was estimated based on input from DoD subject matter experts (SMEs) with visibility into budgeting and therefore reflects actual cost incurred. The remaining direct tier 4 costs were associated with labor including the nurse and laboratory technician time for swab sampling and data management and reporting. We obtained average USAF nurse base salary values from Salary.com, a leading industry source of compensation data [31]. Laboratory technician and USAF general staff salary information were obtained from Indeed [32] and Glassdoor [33], respectively, which are both crowdsourced databases of employers and employees. We assumed a flat benefits rate of 35%, and this was added on top of base compensation values to estimate the staff’s total compensation rates.

WWS labor included a variety of USAF staff and contractors for base and sampling site selection, onboarding bases and training base personnel, obtaining samples, sample processing, data management and reporting, vendor management, and ongoing support to participating bases. The estimate of hourly rate for sample collectors was obtained from publicly available USAF pay tables [34], and contractor rates were estimated based on technical and pricing SME input and informed by relevant historical experience.

### Economic Cost Model

Our analysis addressed the cost of implementing WWS at 82 AFBs, which was the forecasted number of bases expected for a full-scale mature WWS program [35]. The cost of WWS was evaluated relative to implementing tier 4 diagnostic testing for the same AFBs. We developed a spreadsheet model in Microsoft Excel (Version 2302) to calculate and compare total costs for each surveillance protocol across different scenarios. We assumed that there are negligible, if any, startup costs to tier 4 PCR surveillance. We also assume that bases are equipped with suitable resources for tier 4 surveillance since the pandemic spurred those initial investments. The time for staff to obtain a clinical PCR test is calculated as the major source of lost work time for tier 4 surveillance. We assume minimal loss of work under WWS since it does not require time spent out of operational environments for staff to get tested, like in tier 4 diagnostic surveillance. The additional staffing required to administer the WWS program and conduct tests is included in the calculation. The spreadsheet containing the model calculations is provided in Table S1 in Multimedia Appendix 1.

### Cost-Effectiveness Analysis

We modeled several scenarios to explore the potential costs associated with a range of implementation plans and disease outbreak conditions. The study specifically considered a baseline COVID-19 monitoring scenario (scenario 1), an additional scenario that explored constant higher WWS frequency for COVID-19 monitoring (scenario 2), and two scenarios that included increased testing during the 4-month simulated outbreaks (scenarios 3 and 4). For each scenario, the number of outbreak months and number of monthly tests (per base) are described in Table 2. The Air Force tier 4 sentinel surveillance in practice carried out an average of 293 swab tests per base per month, and we assumed that testing would double during outbreaks. The WWS surveillance pilots operated on a once-weekly basis, but some evidence exists supporting the benefits of increased sampling frequency. Therefore, we considered increases in baseline sampling and large increases in sampling during outbreaks.

**Table 2.** Cost analysis model scenarios comparing tier 4 surveillance and wastewater surveillance (WWS).

Scenario		Outbreak months/year, n	Tier 4 swab tests per base per month, n	WWS tests per base per month
COVID-19 monitoring		None	293	4.33 (1/week)
COVID-19 monitoring with higher WWS frequency		None	293	8.66 (2/week)
Outbreak scenario with increased tier 4 testing		4	293 normally; 586 in outbreaks	4.33 (1/week) in all months
Outbreak scenario with increased tier 4 and WWS testing		4	293 normally; 586 in outbreaks	4.33 (1/week) normally; 21.66 (5/week) in outbreaks

### Ethical Considerations

This work does not involve data derived from human or nonhuman animal subjects and does not involve the collection of any new data. Therefore, it does not require ethical approval.

## Results

**Figure 1.**
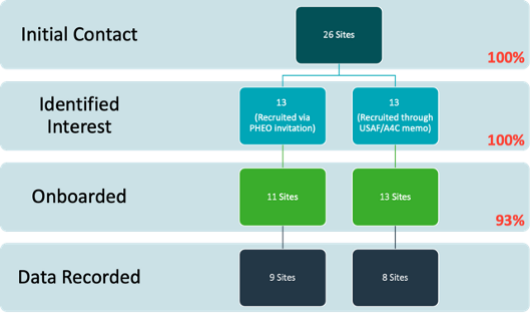
Data collection procedure for 17 of the 26 air force bases that recorded COVID-19 wastewater surveillance data during the period from September 2021 to January 2022. A4C: Air Force Directorate of Civil Engineers; PHEO: public health emergency officer; USAF: US Air Force.

**Figure 2.**
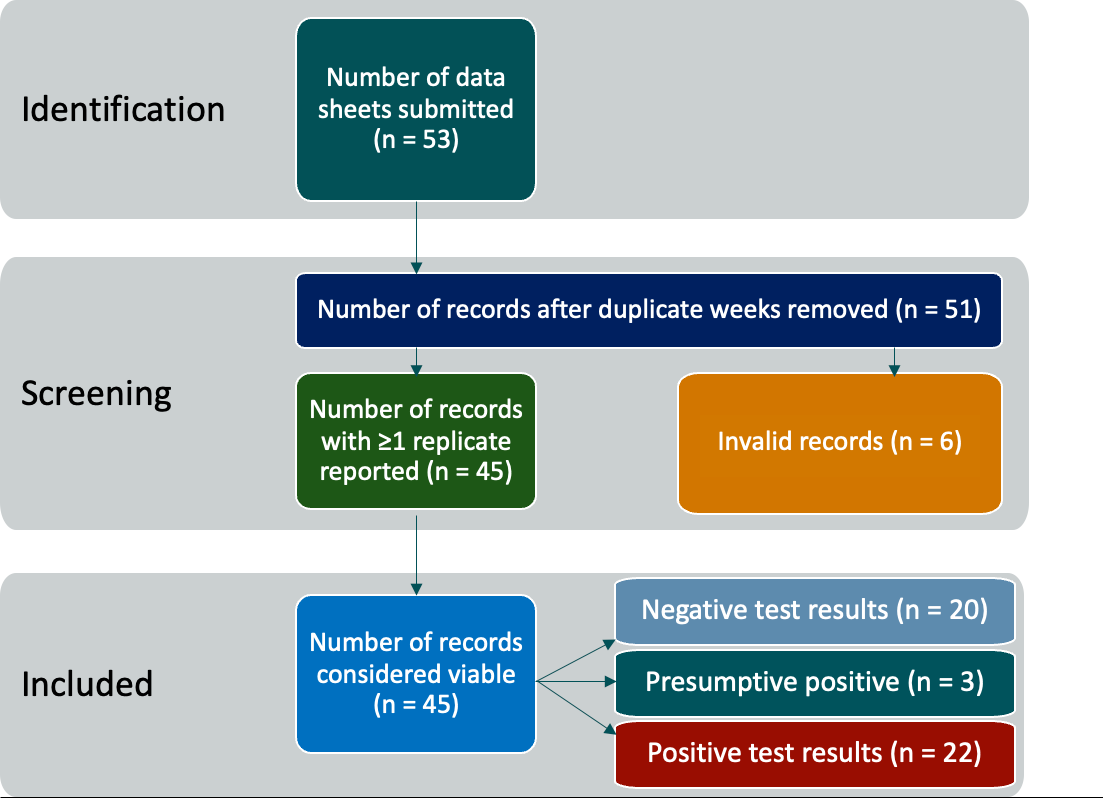
Data preprocessing workflow for the 53 submissions from 17 of 26 air force bases that recorded COVID-19 wastewater surveillance data from September 2021 to January 2022.

Of the 26 AFBs, 17 recorded WWS data during the period from September 2021 to January 2022 (Figure 1), demonstrating that sewage can be safely sampled in a field environment and at a fixed lab. The procedures implemented at sites during the pilot were designed to collect and process wastewater samples once per week. There were 53 data submissions recorded, and those submissions amounted to 45 unique viable sample records. Invalid submissions included duplicate records and samples with PCR reaction issues such as incubation temperature and sample concentration (Figure 2). Three sites were used strictly for an early feasibility pilot stage in which protocols were established. The remaining 14 sites submitted data collected over partially overlapping periods of 4.5 weeks on average. The pilot study identified 25 positive (or presumptive positive) samples and 20 negative samples in total. The 25 positive samples came from 12 of the 14 sites that collected samples systematically. However, the sites did not collect the same number of samples, and 2 sites that detected no positives were also the sites that submitted the fewest total samples. This procedure not only validated the feasibility of implementing WWS at AFBs but also highlighted considerable site-to-site variability in executing systematic sampling procedures. These results provided a case study from which we derive assumptions for the economic cost model.

The cost of SARS-CoV-2 WWS was estimated and compared to the estimated cost of tier 4 COVID-19 sentinel surveillance (asymptomatic testing) across 82 selected AFBs. The four scenarios modeled are described in Table 2. For each scenario, we used the cost model parameters to estimate the total direct and indirect costs of both WWS and tier 4 surveillance. Table 3 shows the total annual costs (in millions of 2021 US dollars) for each scenario at the 82 AFBs. In scenarios 1 and 2, we estimated that the direct costs of the tier 4 sentinel surveillance program would cost approximately US $18 million under baseline COVID-19 monitoring. We estimated that WWS would cost between US $5.4 million with once-weekly testing (scenario 1) to US $7.5 million with twice-weekly testing (scenario 2).

**Table 3.** Cost-effectiveness analysis for wastewater surveillance (WWS) and tier 4 surveillance scenarios.

Scenario	Tier 4 direct cost (US $)	WWS direct cost (US $)	Cost difference (US $)	Cost of lost work (US $)
COVID-19 monitoring	18.0 million	5.4 million	12.5 million	5.6 million
COVID-19 monitoring with higher WWS frequency	18.0 million	7.5 million	10.5 million	5.6 million
Outbreak scenario with increased tier 4 testing	24.0 million	5.4 million	18.5 million	7.5 million
Outbreak scenario with increased tier 4 and WWS testing	24.0 million	8.2 million	15.8 million	7.5 million

Enhanced surveillance is needed to manage the response during an outbreak, here defined broadly as either local- or national-level transmission that is sufficiently high such that strict control measures are put in place. Therefore, under COVID-19 outbreak monitoring scenarios 3 and 4, we estimated the direct costs of tier 4 sentinel surveillance to be US $24 million. The cost of WWS may also go up depending on policy decision-making; for example, sites could choose to test more frequently or utilize alternative pathogen detection methods. In scenario 3, only tier 4 sentinel surveillance is increased during the outbreak response, so the estimated WWS direct costs remain US $5.4 million. In contrast, scenario 4 assumed that the use of both tier 4 sentinel surveillance and WWS go up during the outbreak response, so the estimated WWS direct costs increased to US $8.2 million.

Tier 4 sentinel surveillance PCR testing requires that USAF staff take time to get tested, and this leads to additional costs. We used our model to estimate the cost of lost work, based on typical staff salary ranges and time required to get tested. We estimated that there would be a US $5.6 million cost for the loss of work associated with tier 4 sentinel surveillance PCR testing in scenarios with baseline testing. During a disease outbreak scenario leading to increased testing, we estimated the cost of lost work to be US $7.5 million. In contrast, WWS does not place any burden on staff not associated directly with the implementation of the surveillance program, and thus, we include zero additional costs during outbreaks for those scenarios.

We found that the cost of WWS was between US $10.5-$18.5 million less expensive annually in direct costs when compared to tier 4 sentinel surveillance and that tier 4 sentinel surveillance has an additional cost of US $5.6-$7.5 million annually due to USAF personnel losing time for testing. If WWS were implemented, there would still be the capacity to carry out a substantial amount of tier 4 sentinel surveillance PCR testing. We quantified the break-even point for combined WWS and PCR testing by calculating the number of PCR swab tests that could be conducted per base per month under the WWS paradigm while breaking even with the higher cost of the original tier 4 testing scheme. The results of our break-even analysis are shown in Table 4.

**Table 4.** Break-even swab tests by scenario.

Scenario	Break-even swab tests based on direct cost difference only^a^ (per AFB^b^/month), n	Break-even swab tests including cost of lost work^a^ (per AFB/month), n	
COVID-19 monitoring	204	225	
COVID-19 monitoring with higher WWS^c^ frequency	171	200	
Outbreak scenario with increased tier 4 testing	302	323	
Outbreak scenario with increased tier 4 and WWS testing	258	289	

a Displayed are the number of swab tests per base per month added that can be conducted with WWS at the break-even point relative to tier 4 costs.

b AFB: air force base.

c WWS: wastewater surveillance.

To estimate the break-even point based on direct costs only, we simply took the direct cost differences and divided them by the direct cost per swab test (US $62.39/test for materials and labor), spread across 82 bases and 12 months. Under COVID-19 baseline monitoring with once-weekly WWS (scenario 1), we estimated that an additional 204 swab tests per AFB per month could be added to the WWS protocol for the same cost as the original tier 4 sentinel surveillance scheme. If WWS was increased to twice weekly (scenario 2), then we estimated an additional 171 swab tests could be performed at the break-even point. When the cost of lost work is incorporated, including assumed lost work for swabs used to reach the break-even point, we estimated that 225 and 200 additional swab tests could be performed in scenarios 1 and 2, respectively. The demand for all forms of surveillance increases during an outbreak, so more swab tests can be performed at the break-even cost point. When the cost of lost work is included, we estimated that 323 and 289 additional swab tests could be performed for outbreak scenarios 3 and 4, respectively. We estimated that more than half of the tier 4 sentinel surveillance program could be maintained across all scenarios, while WWS is implemented in parallel with no additional cost (ie, at the break-even point).

## Discussion

The DoD SARS-CoV-2 WWS surveillance pilot studies demonstrated the feasibility of implementing WWS at military installations. The pilot studies revealed some important technical considerations. For example, although dPCR was extremely sensitive, it required shipping of wastewater from remote sites to a centralized location, potentially limiting its use in large-scale deployment. Portable quantitative PCR had a lower throughput of samples than dPCR but was simple to use at the point of sampling. Our simple cost model considered these lessons.

The pilot studies also provided real-world data on the costs associated with WWS when compared to standard swab-based testing, including material costs and labor requirements. In general, WWS for SARS-CoV-2 may offer several benefits, including earlier detection of outbreaks, lower costs and burden for community-wide coverage compared to diagnostic testing, and detection of viral presence regardless of symptoms. Coupling our analysis with the overall results of the DoD pilot studies suggests that those benefits are likely to hold for other DoD use cases. Specifically, our model suggests that the deployment of WWS to AFBs would be substantially more cost-effective than broad asymptomatic swab testing. Our break-even analysis indicated that without allocating additional funding to surveillance efforts, WWS could be implemented for AFB-level monitoring, and swab testing could be used for more targeted purposes or simply in parallel. It is important to note that swab testing and WWS do not provide the same information. Swab testing can enable individual-level actions, such as quarantining and contract tracing, and higher resolution data. Therefore, trade-offs between the public health benefits of WWS and swab testing must be determined case by case. These findings apply to both baseline COVID-19 monitoring and scenarios where outbreaks are occurring on bases throughout the year.

Our cost model was intentionally simplistic to enhance transparency for decision makers. That simplicity is also a limitation in that there may be unforeseen complexities and costs associated with scaling the WWS program beyond the pilot sites. In addition, our cost data is primarily derived from the USAF WWS pilot program, and facilities associated with other branches of the DoD may require different considerations. Many of our parameter estimates were obtained from SMEs (ie, individual DoD staff and contractors associated with the pilot studies) as opposed to independent reviews of pilot study budgets. Therefore, our cost estimates should not be interpreted as formal financial forecasts.

Another limitation of our analysis is the lack of an uncertainty estimation. Any formal program-level financial forecast would require uncertainty ranges to be estimated in addition to cost estimates. Many of the material costs were obtained directly from individuals with knowledge of the actual costs incurred during the pilot studies for this work. Therefore, our model could be framed as an estimate of what the actual cost would have been if the pilot was carried out at all AFBs rather than a forecast of the costs of a DoD-wide program—though, we believe our work is germane to that topic. Furthermore, systematic uncertainty in labor and materials costs due to changes in supply chain issues and inflation are likely correlated such that a proper uncertainty propagation would require estimating the joint distribution of costs, which is beyond the scope of our efforts. Given the magnitude of the point difference and the consensus in the literature that WWS is less expensive for population-level monitoring—albeit not necessarily cost-effective if implemented poorly [25]—we believe that our results are qualitatively robust to underlying uncertainty in the data and model specification.

In conclusion, we found that the USAF WWS pilot was a cost-effective complement to standard swab-based testing in the tier 4 sentinel surveillance program. We believe that WWS in tandem with swab-based testing is the best approach to maximize available resources. Looking beyond the COVID-19 pandemic, the DoD can be an important partner in global pandemic and all-hazard preparedness efforts. WWS is uniquely well suited to multi-threat biological surveillance, and our results suggest that the adoption of WWS across US military installations would help deliver a more comprehensive early warning system. A fully developed WWS program would complement civilian efforts like the National Wastewater Surveillance System and enable rapidly scalable outbreak monitoring in the event of future disease outbreaks.

## Supplementary material

10.2196/54750 Multimedia Appendix 1 Cost model and parameters.

## Abbreviations

AFB: air force base
DoD: Department of Defense
dPCR: digital polymerase chain reaction
JPEO-CBRND: Joint Program Executive Office for Chemical, Biological, Radiological and Nuclear Defense
PCR: polymerase chain reaction
SME: subject matter expert
USAF: US Air Force
WWS: wastewater surveillance
